# Genome-Wide Identification and Expression Analysis of the *AP2/ERF* Transcription Factor Gene Family in Hybrid Tea Rose Under Drought Stress

**DOI:** 10.3390/ijms252312849

**Published:** 2024-11-29

**Authors:** Xinyu Yan, Wei Huang, Cheng Liu, Xuan Hao, Chengye Gao, Minghua Deng, Jinfen Wen

**Affiliations:** 1 Faculty of Architecture and City Planning, Kunming University of Science and Technology, Kunming 650021, China; yanxinyu20000812@163.com (X.Y.); huangwei_kmu@163.com (W.H.); haoxuan031300@163.com (X.H.); gaochengye0921@163.com (C.G.); 2 Key Laboratory of Vegetable Biology of Yunnan Province, College of Landscape and Horticulture, Yunnan Agricultural University, Kunming 650201, China

**Keywords:** hybrid tea rose, *RhAP2/ERF*, phylogenetic analysis, differential expression, drought stress

## Abstract

Drought stress is an important factor that reduces plant biomass production and quality. The *APETALA2/ETHYLENE RESPONSE FACTOR* (*AP2/ERF*) gene family is widely involved in biological processes such as plant growth, development, and stress response. However, the characteristics of the *AP2/ERF* gene family in hybrid tea rose (*Rosa × hybrida*) and their potential functions in responding to drought stress are still unclear. In the current study, 127 *AP2/ERF* genes were identified in hybrid tea rose. Phylogenetic analysis showed that the corresponding 127 AP2/ERF transcription factors belonged to five subfamilies. There was a large number of *cis*-acting elements in the *AP2/ERF* gene promoters related to regulation of stress response, growth and development. By examining the RNA sequencing data in the PlantExp database, the *RhAP2*/*ERF* genes exhibiting tissue-specific and stress-responsive expression in rose were identified. Furthermore, three candidate *RhAP2/ERF* genes (*RhDREB36*, *RhERF59*, and *RhDREB44*) that might participate in drought response were determined via qRT-PCR analysis in rose cultivars under drought treatment. Subcellular localization analysis revealed that *RhDREB44* was located in the nucleus. These results provide a foundation for exploring the regulatory functions of *RhAP2/ERF* genes in the growth and development of roses, as well as for selecting key genes for future molecular breeding.

## 1. Introduction

One of the largest families of transcription factors found in plants is the *APETALA2/ETHYLENE RESPONSE FACTOR* (*AP2/ERF*) gene family. It has significant potential applications in plant genetic improvement and breeding [1,2] and is heavily involved in regulating plant growth, development, and stress response. One or two highly conserved AP2 domains are typically found in members of the AP2/ERF transcription factor protein family. Proteins with AP2 domains regulate target genes by binding to specific DNA sequences [3,4]. The AP2 domain consists of 60 to 70 amino acids and has a typical helix-turn-helix structure. The AP2/ERF protein family can be broken down into five subfamilies based on the number of AP2 domains and their characteristic sequences [5]. These subfamilies are called AP2, ERF, DREB, RAV, and Soloist. Most of the time, members of the AP2 subfamily have two AP2 domains and play important roles in controlling how plants grow [6]. ERF, DREB, and RAV subfamily individuals have only one AP2 domain, while RAV subfamily individuals additionally have one B3 domain [7]. The Soloist subfamily includes members with distinctive gene structures and similar AP2 domains [8].

The transcription factors of the AP2 subfamily are involved in different developmental processes of plants. In Arabidopsis, *AP2* regulates flowering time [9,10] and determines seed quality and size [11], while *BBM* can promote somatic embryogenesis [6,12,13,14]. The *ERF* and *DREB* subfamily genes are related to responses to biotic and abiotic biological stresses [15]. The *OsDREB* gene in rice is associated with plant tolerance to drought, high-salt, and low-temperature stress [16]. Four *ERF* genes, including *AtERF6* in Arabidopsis, play important roles in response to strong light [17]. The *RAV* subfamily genes are believed to play a key role in responding to biotic and abiotic stresses, such as Arabidopsis *AtRAV1*, which is related to the ABA (abscisic acid) signaling pathway, and is involved in regulating plant responses to abiotic stresses, such as drought and salinity. Tomato *SlRAV1* is involved in regulating fruit ripening, color changes, and other developmental processes, and responds to biotic stresses (such as pathogens) and abiotic stresses (such as drought and high temperatures) [18,19]. Members of the *AP2/ERF* gene family have been identified in plants like *Arabidopsis thaliana* [5], *Vitis vinifera* [7], *Raphanus sativus* [20], *Zingiber officinale* [21], *Arachis hypogaea* [22], *Oryza sativa* [23], *Saccharum officinarum* [24], and *Zea mays* [25] as more and more plant genomes are sequenced.

*Rosa* × *hybrida* (hybrid tea rose), belonging to the Rosaceae family, is a type of evergreen to semi-evergreen dwarf shrub. The *Rosa* genus originated in China, which is rich in *Rosa* genetic resources [26], mainly distributed in mountainous areas of provinces such as Hubei, Sichuan, and Gansu. *R.* × *hybrida* is widely cultivated as a garden plant worldwide because of its tolerance of a range of conditions and the number and color range of flowers produced. *R. × hybrida* is impacted by various biotic and abiotic stressors, including pests and diseases, high-temperature, salt, and drought stresses, all of which significantly hinder the growth and progress of the *Rosa* × *hybrida* as a popular garden plant [27]. The *TIFY*, *PP2C*, and *MYB* gene families have been systematically and comprehensively analyzed in *R. × hybrida*. More *AP2/ERF* genes in rose will be identified when the technology for gene structure annotation is updated. The structure, phylogenetic relationships, and *cis*-acting elements of the hybrid tea rose *AP2/ERF* gene family were thoroughly examined in the current study. Using transcriptomic data, the expression patterns of *AP2/ERF* genes in various rose tissues and in response to drought treatment were analyzed, and key functional genes that responded to drought stress were identified using quantitative reverse transcription PCR (qRT-PCR). The subcellular distribution of important proteins that are encoded by members of the *AP2/ERF* gene family was also investigated. This study’s findings provide a solid biological foundation for gaining a deeper comprehension of the functions of the rose *AP2/ERF* gene family.

## 2. Results

### 2.1. Identification and Chromosomal Localization of AP2/ERF Genes in Hybrid Tea Rose

**Figure 1:**
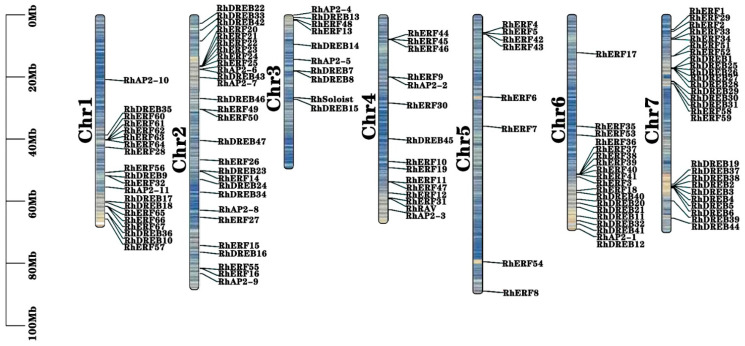
Chromosome localization of *RhAP2/ERF* gene family members in *Rosa × hybrida*. The scale on the left is measured in megabases. The blue to yellow color range inside the chromosome indicates an increase in gene density. The chromosome number is displayed on the left side of the vertical bar; The gene location is displayed on the right side of the vertical bar.

A Blastp search on the *R. × hybrida* whole-genome protein sequences was conducted using 147 Arabidopsis AtAP2/ERF protein sequences as query sequences to identify all members of the protein family. The histone domain’s hidden Markov model (HMM) file, PF00847, was obtained from the Pfam protein family database. All protein sequences were aligned with these HMMs using TBtools software, and incomplete sequences and candidate sequences without the corresponding characteristic domains were excluded from the Pfam, SMART (Simple Modular Architecture Research Tool), and CDD (Conserved Domains Database) databases. mitochondria, and RhDREB14 and RhDREB42 located in the cytoplasm. TBtools software was also used to analyze the chromosomal locations of *RhAP2/ERF* family genes (Figure 1), and the results showed that most of them were located in high-gene-density positions. Chromosome chr2 had the most *RhAP2/ERF* genes (28), while chromosome chr5 had the fewest, only eight. Numbered according to their positions on the 7 rose chromosomes, 127 members of the RhAP2/ERF gene family were identified from the rose genome (Table S1).

The length of RhAP2/ERF sequences encoded varied from 125 to 832 amino acids, with isoelectric point (pI) values predicted to range from 4.52 to 10.28 and molecular weights predicted to vary from 13,942.8 kDa to 91,007.8 kDa. The RhAP2/ERF proteins were predicted to be mainly located in the nucleus, with other predictions being 17 RhAP2/ERFs located in the chloroplasts, RhDREB23, RhDREB37, and RhERF2 located in the mitochondria, and RhDREB14 and RhDREB42 located in the cytoplasm.

### 2.2. Phylogenetic Analysis and Multiple Sequence Alignment of AP2/ERF Genes in Hybrid Tea Rose

**Figure 2:**
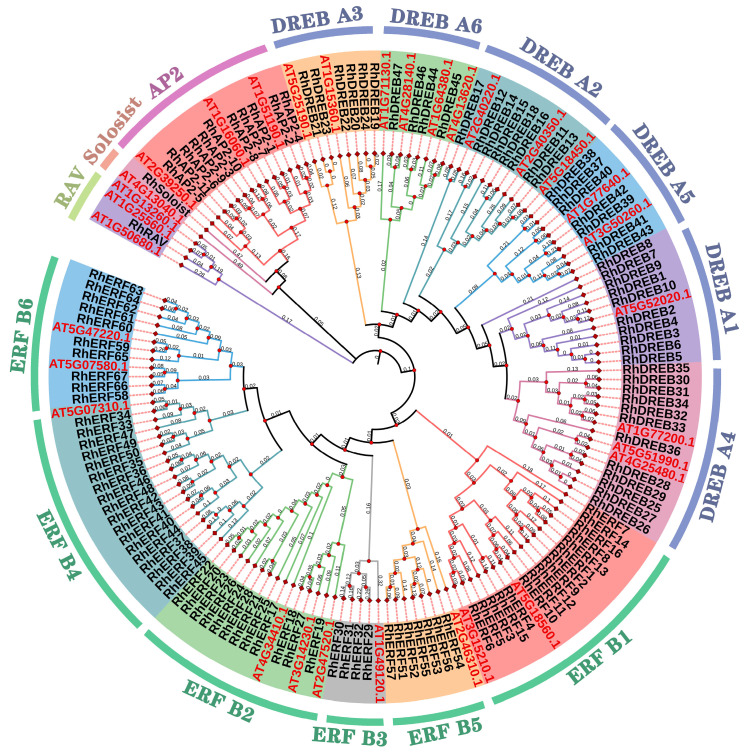
Phylogenetic analysis of AP2/ERF proteins in *Rosa × hybrida* and *Arabidopsis thaliana*. Using MEGA7.0 software, a rootless adjacency phylogenetic tree was constructed using the AP2/ERF domains of the AP2/ERF proteins in *R. × hybrida* and *A. thaliana*, and 1000 bootstrap support analyses were conducted. The figure shows five subfamilies: I, II, III, IV, and V, with subtypes represented by different colored regions.

Using the Arabidopsis *AP2/ERF* gene family as a reference, a phylogenetic tree of rose and Arabidopsis AP2/ERF protein sequences was constructed by the rootless adjacency method (Figure 2). The results showed that these transcription factors could be divided into five subfamilies, namely AP2, ERF, DREB, RAV, and Soloist, and could be further divided into 15 evolutionary branches. The AP2, DREB, and ERF subfamilies contained 11, 48, and 67 members, accounting for 8.66%, 37.79%, and 52.76% of the total members, respectively, whereas the RAV and Soloist subfamilies contained only one member each. Based on studies on plants such as Arabidopsis [2], the DREB subfamily could be further divided into six subtypes, namely A1–A6. In roses, members of this subfamily were concentrated in subtypes A1 and A4, with A1 containing 10 members and A4 containing 12 members. The ERF subfamily could be further divided into 6 subtypes, namely B1–B6, with these 6 subtypes of the rose ERF subfamily containing 16, 12, 4, 18, 7, and 10 members, respectively.

**Figure 3:**
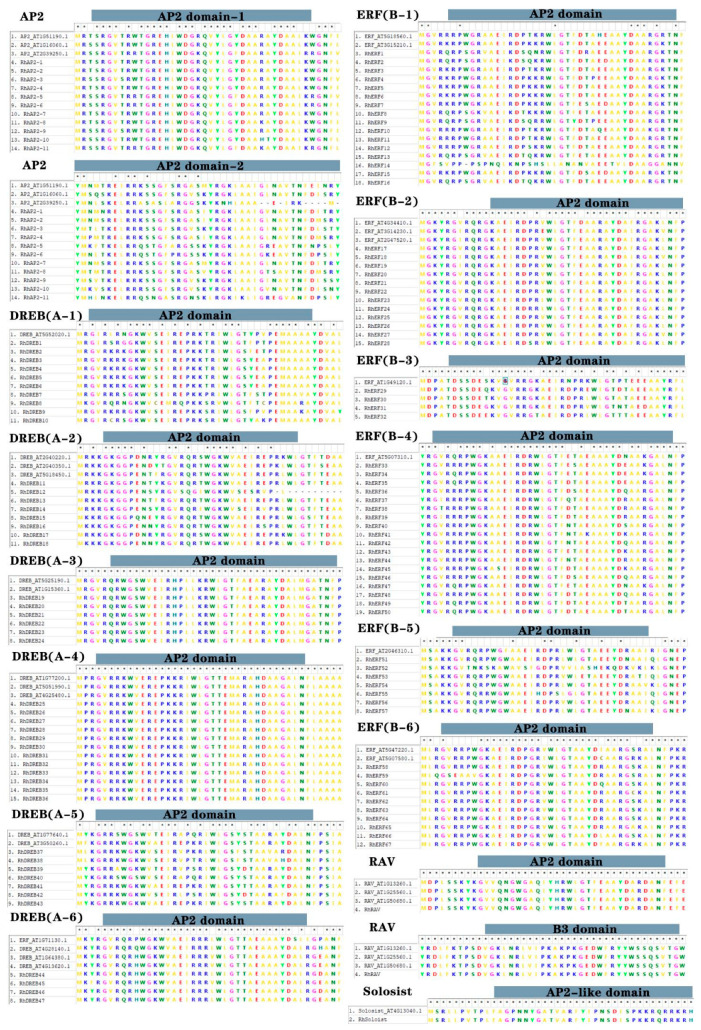
Multiple sequence alignment of AP2/ERF proteins in *Rosa × hybrida* and *Arabidopsis thaliana*. Using the ClustalW tool of MEGA7.0 software, multiple sequence alignment was performed on 127 RhAP2/ERF and 32 AtAP2/ERF amino acid sequences using the AP2 domain of AP2/ERF proteins from *R. × hybrida* and *A. thaliana*, and 1000 bootstrap support analyses were performed. The figure shows five main subfamilies: AP2, ERF, DREB, RAV, and Soloist. * indicates consistency.

The multiple sequence alignment of the AP2/ERF protein family members via the ClustalW tool in MEGA7 (v7.0.14) software showed a high degree of similarity among them. The positions of the intermediate regions were highly similar, and they contained the conserved structural domains of the AP2/ERF protein family (Figure 3). Among the 127 RhAP2/ERF proteins, most of the AP2/ERF domains were highly conserved, with AP2 subfamily members having two AP2 domains, while ERF, DREB, and RAV subfamily members each had only one AP2 domain. RAV subfamily members had one AP2 domain and one B3 domain, while Soloist subfamily members had domains that were similar to AP2. In summary, the AP2 subfamilies are mostly composed of two AP2 conserved domains, while the ERF, DREB, RAV and Soloist subfamilies are mostly composed of one AP2 conserved domain.

The multiple sequence alignment of the AP2/ERF protein family members via the ClustalW tool in MEGA7 software showed a high degree of similarity among them. The positions of the intermediate regions were highly similar, and they contained the conserved structural domains of the AP2/ERF protein family (Figure 3). Among the 127 RhAP2/ERF proteins, most of the AP2/ERF domains were highly conserved, with AP2 subfamily members having two AP2 domains, while ERF, DREB, and RAV subfamily members each had only one AP2 domain. RAV subfamily members had one AP2 domain and one B3 domain, while Soloist subfamily members had domains that were similar to AP2. In summary, the AP2 subfamilies are mostly composed of two AP2 conserved domains, while the ERF, DREB, RAV and Soloist subfamilies are mostly composed of one AP2 conserved domain.

### 2.3. Motifs, Gene Structures, and Domains of AP2/ERF Genes in Hybrid Tea Rose

**Figure 4:**
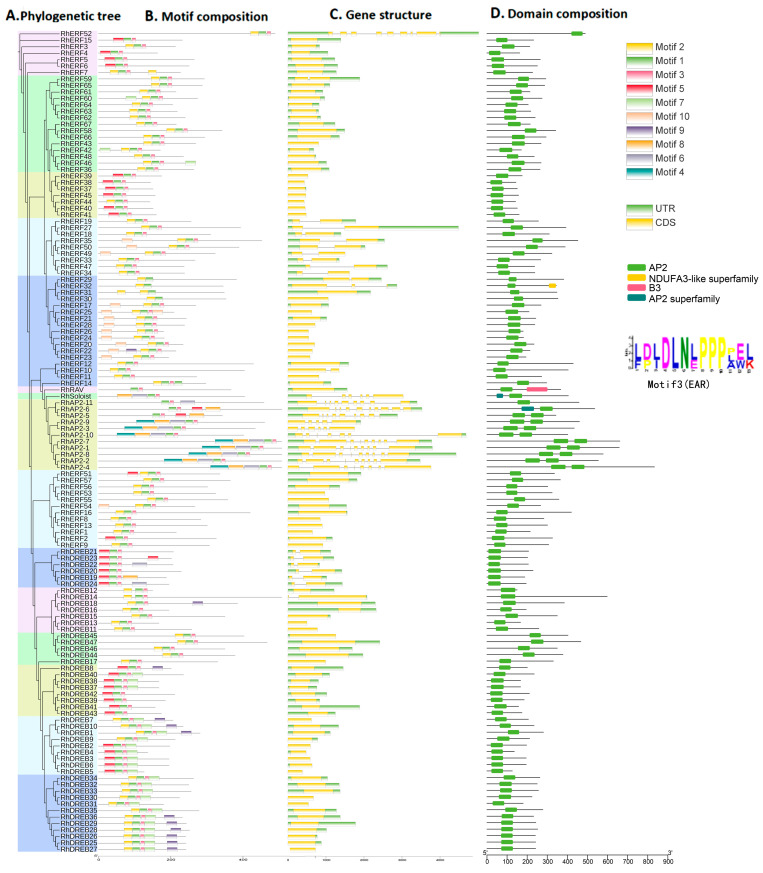
Phylogenetic tree, motif composition, gene structure, and domain composition of RhAP2/ERF: (**A**) Phylogenetic tree of RhAP2/ERF protein. (**B**) The motif composition of RhAP2/ERF. (**C**) The gene structure of *RhAP2/ERF*. (**D**) Domain composition of the *RhAP2/ERF* gene.

Based on the simplified phylogenetic tree constructed, 127 RhAP2/ERF proteins were divided into 5 groups and 15 subgroups (Figure 4A). Analysis of the conserved motifs of the *AP2/ERF* transcription factor genes in rose (Figure 4B) showed that 80 members of the *AP2/ERF* gene family in rose simultaneously possessed Motif1, Motif2, and Motif3, suggesting that these three motifs are the main ones making up the AP2 domain. Motif 3 is known as the ethylene responsive factor (ERF)-associated amphiphilic repression (EAR) motif, with a conserved amino acid sequence of L/FDLNL/F (X) P, which can inhibit the intermolecular activity of other transcription activators. Except for *RhAP2-5*, *RhAP2-6*, and *RhAP2-11*, all other members of the AP2 family contained Motif 1, Motif 3, Motif 4, Motif 6, and Motif 8. The *Harv* gene had only Motif 2 and Motif 3, while most members of the *DREB* subfamily contained Motif 7, and only a small number of members contained Motif 9. In the *ERF* subfamily, only *RhERF42* and *RhERF46* contained Motif 7.

There were significant differences in the gene structure between the various *AP2/ERF* subfamilies of rose (Figure 4C). All *AP2* subfamily genes contained multiple introns (between five and ten). Except for *RhERF52*, which contained eight introns, the other 66 members of the *RhERF* subfamily and all members of *RhDREB* had either one or two introns. *RhRAV* had one intron, while *RhSoloist* had six introns.

Protein molecules can contain multiple structurally specific and functionally distinct regions or domains. Analysis of conserved domains in the RhAP2/ERF protein structure revealed four types of conserved domains, namely the AP2, the NDUFA3-like superfamily, B3, and the AP2 superfamily (Figure 4D). The members of the rose AP2/ERF protein family all had one or two AP2 domains, indicating that the AP2 domain is the main functional component. In the AP2 subfamily, RhAP2-11 contained one AP2 domain, while all other members of this subfamily had two AP2 domains. All members of the DREB subfamily possessed one AP2 domain. Except for RhERF32 (which contains one AP2 domain and one NDUFA-like superfamily domain), all members of the ERF subfamily had only one AP2 domain. The members of the RAV subfamily had one AP2 domain and one B3 domain, while the Soloist subfamily had two AP2 domains.

In summary, these results indicate that different categories of *RhAP2/ERF* genes may have evolved different functions and there is a certain degree of differentiation in the intron/exon structure.

### 2.4. The Cis-Acting Elements in Promoters of the AP2/ERF Gene Family in Hybrid Tea Rose

**Figure 5:**
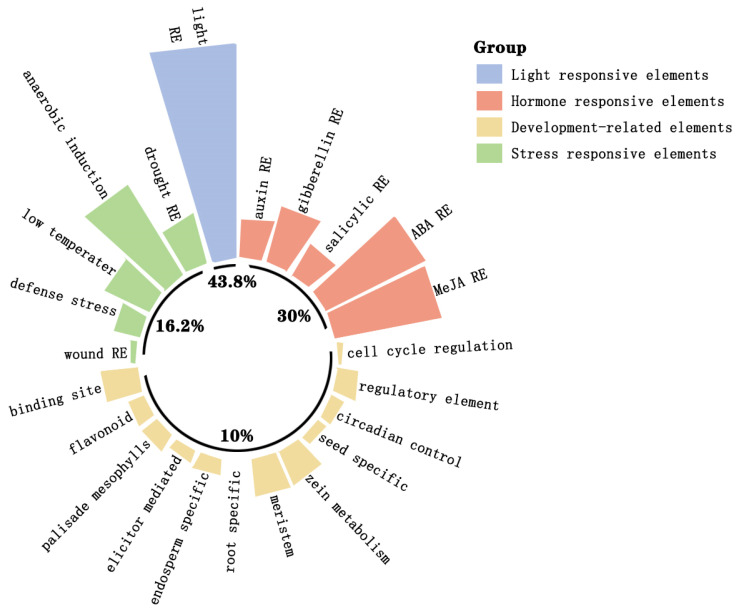
Types of *cis*-elements in the *RhAP2/ERF* gene promoter of *Rosa × hybrida* and the distribution of quantities. RE = responsive element.

The *cis*-acting elements in the promoter region play an important role in regulating gene expression. Using the PlantCARE database, the *cis*-acting elements of the *AP2/ERF* gene family promoter region in rose were analyzed. A total of 3561 specific elements were detected, which could be divided into four categories: light-responsive, hormone-responsive, stress-responsive, and development-related elements. Among them, hormone-responsive elements involved response to gibberellin, abscisic acid, auxin, methyl jasmonate, and other hormones, stress-responsive elements involved response to hypoxia, drought, low temperature, defense, and other related factors, and development-related elements involved response by endosperm and meristematic tissues (Figure S1). Light-responsive (1558), abscisic acid-responsive (410), methyl jasmonate-responsive (393), and drought-induced (92) elements proved to be the main *cis*-acting elements of the *AP2/ERF* gene promoter sequence in rose. Among them, light-responsive elements were present in all the gene promoters, indicating that the *AP2/ERF* genes in rose may be involved in plant photomorphogenesis or light-related environmental adaptation (Figure 5). The number of *AP2/ERF* genes in rose with hormone-responsive elements, involving hormones such as methyl jasmonate, abscisic acid, gibberellins, or auxins, were 95, 100, 74, and 57, respectively, indicating that members of the rose *AP2/ERF* gene family are widely involved in different plant hormone-signaling pathways. In addition, *RhERF6*, *RhERF23*, *RhERF24*, and *RhDREB10* contained stress-responsive elements, while *RhDREB41*, *RhERF4*, *RhERF46*, and *RhERF62* had cell cycle regulatory elements. Those genes with specific elements potentially have more specific functions.

### 2.5. Collinearity Analysis of AP2/ERF Genes in Hybrid Tea Rose

**Figure 6:**
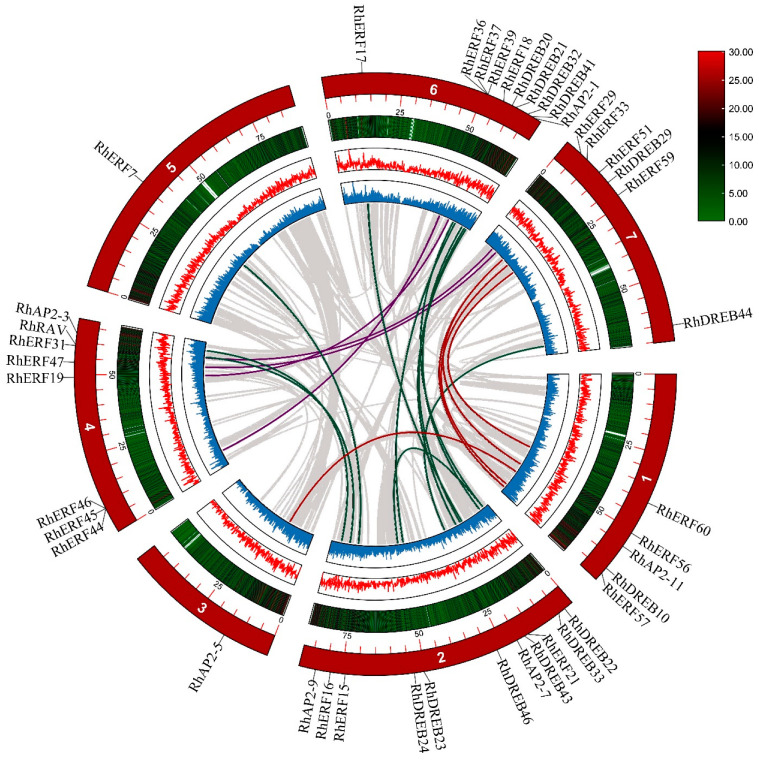
Gene replication of the *AP2/ERF* gene family in *Rosa × hybrida*. The colored lines represent replicated gene pairs in *RhAP2/ERF*, while the gray lines represent collinear gene pairs in the genome.

MCScanX was used to analyze gene duplication events among the *RhAP2/ERF* genes. There were 22 pairs of duplicated genes in the *RhAP2/ERF* genes (Figure 6 and Table S2), all of which are segmental duplications, with no tandem duplications. The *RhERF51* and *RhDREB22* genes had undergone multiple duplications. In addition, we calculated the non-synonymous (Ka) and synonymous (Ks) substitution rates, as well as the Ka/Ks ratio (Table S2), to capture the evolutionary dynamics of the *RhAP2/ERF* protein-coding sequences. Among them, 17 pairs of duplicated genes had Ka/Ks values less than 1, indicating that these genes mainly underwent purifying (negative) selection, in which detrimental alleles were removed.

### 2.6. Collinearity Analysis of AP2/ERF Genes in Hybrid Tea Rose and Other Species

**Figure 7:**
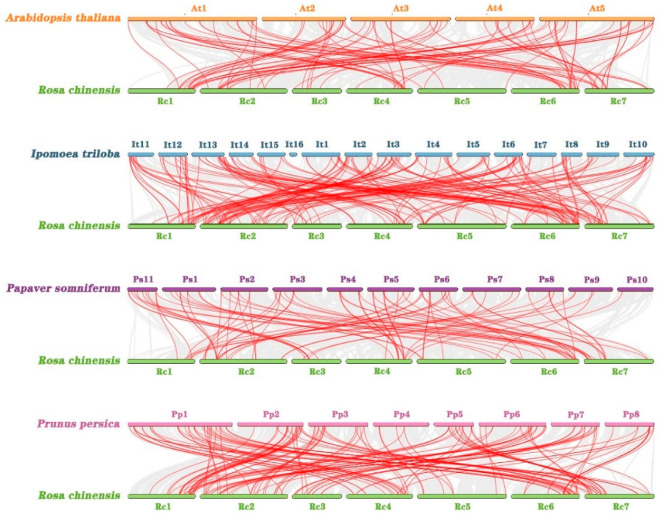
Collinear analyses of *AP2/ERF* genes between *Rosa × hybrida* and four plants (*Arabidopsis thaliana*, *Ipomoea triloba*, *Papaver somniferum*, and *Prunus persica*). The gray lines between rose and other plants represent collinear blocks in wide regions of the genomes, while red lines show the orthologous relationships of *AP2/ERF* genes.

In order to explore the evolutionary relationship between *RhAP2/ERF* genes in hybrid tea rose and other species, collinearity analysis was performed on the genomes of four other plant species (*A. thaliana*, *Ipomoea triloba*, *Papaver somniferum*, and *Prunus persica*), as well as rose. There were 94, 165, 86, and 142 pairs of segmental duplications between *R. × hybrida* and *A. thaliana*, *I. triloba*, *P. somniferum*, and *P. persica*, respectively (Figure 7).

### 2.7. Protein–Protein Interaction Network Analysis of AP2/ERF Proteins in Hybrid Tea Rose

**Figure 8:**
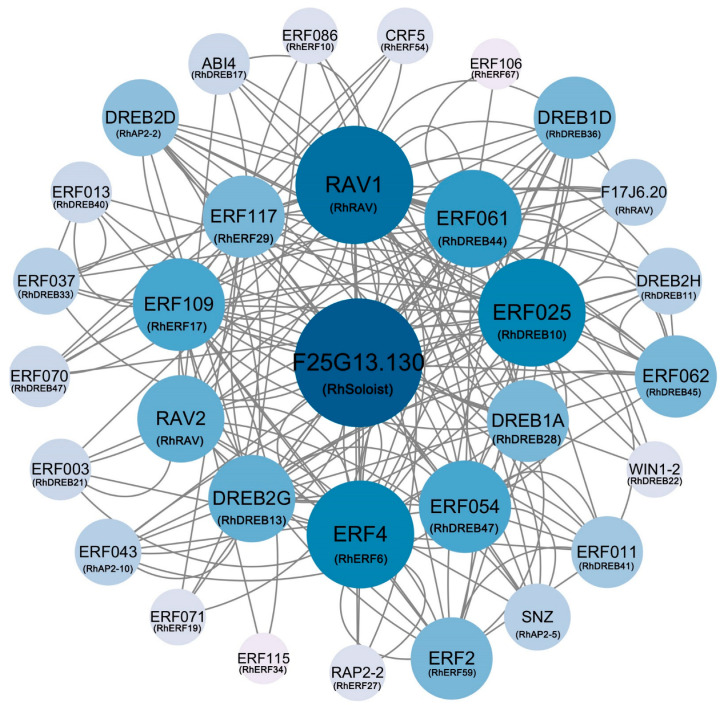
AP2/ERF protein interaction network in roses, visualized using Cytoscape (v3.10.0) software. The blue ball (node) represents the *RhAP2/ERF* gene. The connecting lines represent potential regulatory relationships, and the text displays the predicted gene names. The larger the size and darker the color of the ball, the more it interacts with other proteins.

In order to analyze any synergistic effect of AP2/ERF transcription factors in the regulation process of rose, an interaction network was constructed using 32 Arabidopsis homologous proteins matched from the STRING database (Figure 8). The results showed that RhAP2/ERF proteins with high sequence similarity to AtERF061 (RhDERE44), AtF25G13.130 (RhSoloist), AtRAV1 (RhRAV), AtERF4 (RhERF6), AtERF109 (RhERF17), and AtERF025 (RhDREB10) were the core nodes of the protein–protein interaction network, indicating that these six genes may play an important role in biological functions. In addition, there were complex interactions within and between members of each subfamily, such as RhSoloist, RhERF6, and RhDREB28 forming a triangular interaction network, while the core member RhSoloist interacted with multiple ERF and DREB members, and RhRAV also interacted with multiple ERF and DREB members. The complex interactions between members of different subfamilies suggest that these genes may have very complex functional interactions.

### 2.8. Expression Levels of AP2/ERF Genes in Different Hybrid Tea Rose Tissues and Under Drought Stress Treatments

**Figure 9:**
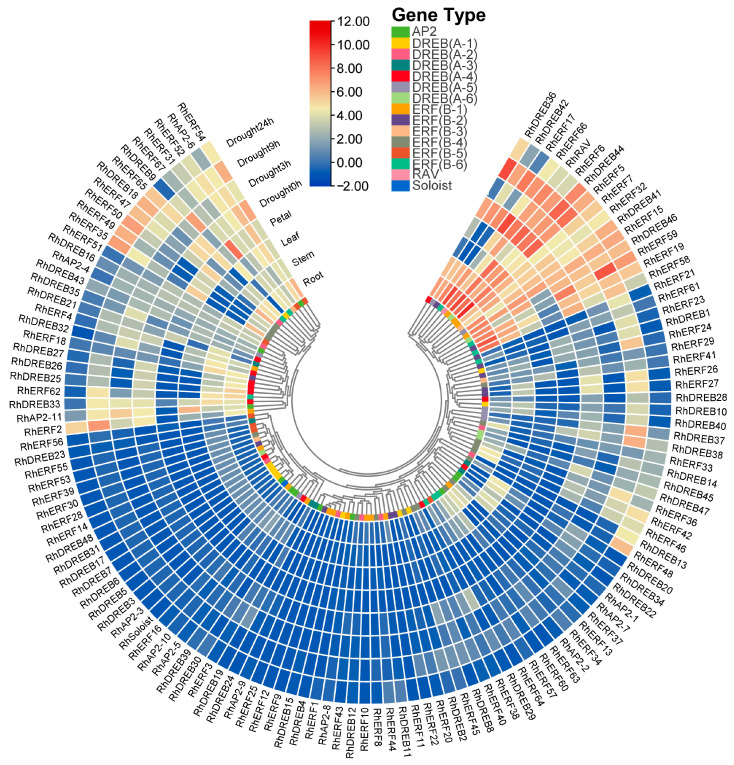
Heat map of *AP2/ERF* gene expression in *Rosa × hybrida* in different tissues and under different durations of drought treatments. The colors from blue to red represent different levels of expression. Blue represents low expression, red represents high expression.

Using transcriptome data downloaded from the PlantExp database, the expression data on 127 members of the *AP2/ERF* gene family in rose roots, stems, leaves, and petals were analyzed, and the results showed that the expression of 122 of the genes had strong tissue specificity (Figure 9). Eight genes (*RhERF5*, *RhERF6*, *RhERF66*, *RhDREB44*, *RhERF67*, *RhERF15*, *RhERF7*, and *RhRAV*) showed high expression levels in different tissues, indicating that these genes play a fundamental role in plant growth and development. Of these genes, *RhERF15*, *RhERF5*, *RhERF6*, and *RhDREB 44* were most actively expressed in the leaves, and *RhERF5*, *RhERF6*, *RhERF66*, and *RhDREB44* had the highest expression in roots, while *RhERF7* and *RhERF67* had the highest expression in stems and flowers, respectively.

Moreover, RNA sequencing (RNA-seq) data retrieved from the PlantExp database were utilized to investigate the transcriptional patterns of RhAP2/ERF genes in response to drought treatments. Of the 127 members of the *AP2/ERF* gene family, 122 genes were expressed in leaves, including 11 *AP2* subfamily genes, 46 *DREB* subfamily genes, *64 ERF* subfamily genes, one *RAV* subfamily gene, and one *Soloist* subfamily gene (Figure 9). Further differential expression analysis showed that eight genes (*RhDREB36*, *RhERF59*, *RhDREB44*, *RhERF17*, *RhERF2*, *RhERF35*, *RhERF50*, and *RhDREB37*) exhibited high expression levels under drought stress compared with control (no-stress, 0 h drought; Figure 9) leaves. These results suggest that *ERF* and *DREB* subfamily genes may play important regulatory roles in the response to drought stress in rose.

**Figure 10:**
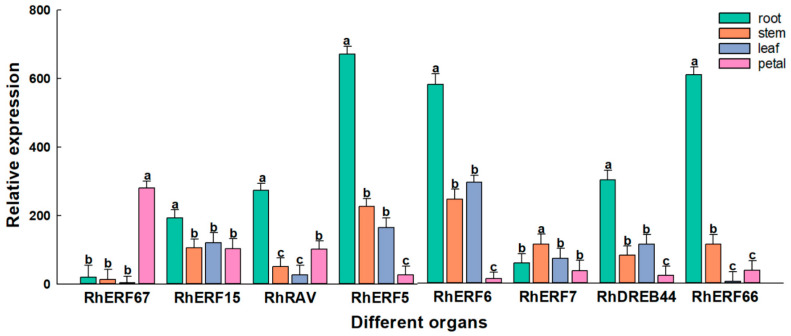
Relative expression levels of eight *RhAP2/ERF* genes were determined by qRT-PCR in different rose organs. Data represent the means ± SEs of three reproducible experiments. Bars with lowercase letters above the columns indicate significant differences between the four organs at *p* < 0.05 (Tukey’s test).

To verify the transcriptome data, eight *RhAP2/ERF* genes with high expression levels (*RhERF5*, *RhERF6*, *RhERF7*, *RhERF15*, *RhERF66*, *RhERF67*, *RhDREB44*, and *RhRAV*) according to transcriptomics were selected and their expression levels were measured in roots, stems, leaves, and petals, using qRT-PCR (Figure 10). It is obvious that the expression levels of these genes vary greatly in different organs. Most of these genes had the highest expression levels in roots, except for *RhERF7*, which had the highest expression in stems, and *RhERF67*, which had the highest expression in petals. The gene expression levels of *RhERF5* and *RhERF66* showed the greatest differences among different organs. They had the highest expression level in roots, close to 700, while their expression levels were the lowest in petals and leaves, respectively.

**Figure 11:**
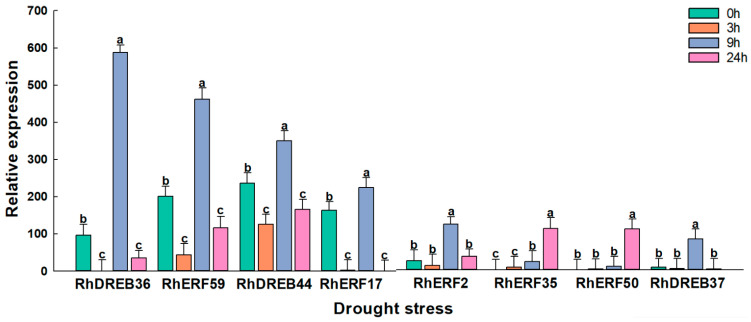
Relative expression levels of 8 *RhAP2/ERF* genes in *Rosa × hybrida* leaves were determined using qRT-PCR after 0 h (CK), 3 h, 9 h, and 24 h of drought stress treatment. Data represent the means ± SEs of three reproducible experiments. Bars with lowercase letters above the columns indicate significant differences between the different treatments at *p* < 0.05 (Tukey’s test).

To screen for *RhAP2/ERF* genes involved in drought stress response, we used qRT-PCR to verify the expression levels of eight *RhAP2/ERF* genes identified from transcriptomic data in leaves as having high expression levels under drought stress (Figure 11). The expression levels of these genes showed significant differences at different durations of drought treatment. In response to drought treatment, the expression of *RhDREB36*, *RhERF59*, *RhDREB44*, and *RhERF17* decreased at 3 h, but gradually increased as the stress period increased, reaching a peak at 9 h. Compared to the 0 h control, the relative expression level of RhDREB36 gene increased fivefold at 9 h, from 100 to nearly 600.

### 2.9. Subcellular Localization of RhDREB44 in Hybrid Tea Rose

**Figure 12:**
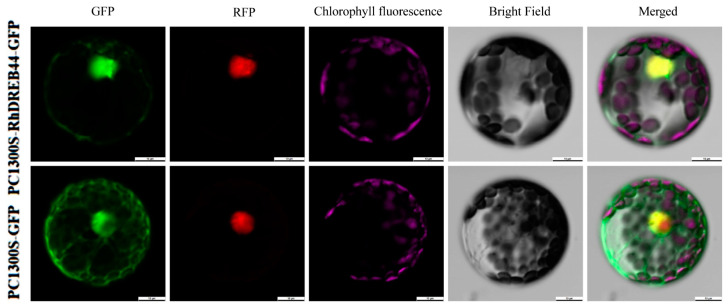
Subcellular localization of RhDREB44 protein in *Arabidopsis* protoplasts. From left to right are *RhDREB44-GFP* green fluorescent protein, *RhDREB44-RFP* red fluorescent protein, chlorophyll fluorescence, bright field, and merged overlay photos (scale bar = 10 μm).

The expression levels of *RhDREB44* in leaves and under drought stress were high. To further investigate the characteristics of *RhDREB44*, subcellular localization analysis was conducted (Figure 12). The positive control PC1300S-GFP exhibited strong green fluorescence in various parts of the Arabidopsis protoplasts, while the green fluorescent protein (GFP) fused to the RhDREB44 protein appeared in the nucleus, indicating that the RhDREB44 protein was localized in the nucleus.

## 3. Discussion

The *AP2/ERF* gene family is one of the largest transcription factor gene families in plants, playing an important role in growth, development, and stress response [2,15]. An important feature of the proteins encoded by the *AP2/ERF* gene family is the conserved AP2 domain, consisting of a 60 to 70 amino acid sequence. The current study identified 127 *AP2/ERF* genes in the rose genome. Similar to the situation in other plant species, these rose *AP2/ERF* genes could be divided into five subfamilies: *AP2*, *ERF*, *DREB*, *RAV*, and *Soloist*, consisting of 11, 48, 67, 1, and 1 gene, respectively. Usually, in plants, the *ERF* subfamily has the most members, followed by *DREB* and *AP2*, with the *RAV* and *Soloist* subfamilies having the fewest members. The numbers of *AP2/ERF* gene subfamily members in rose and other plant species, such as Arabidopsis [15], rice [15], maize [25], and *Populus trichocarpa* [28], exhibit similar patterns, indicating that *AP2/ERF* genes may have a common origin during evolution.

Similar to other species such as *Arabidopsis* [15] and *pecan* [29], all members of the *RhAP2/ERF* gene family were classified into five major categories based on phylogenetic relationships: AP2, ERF, DREB, RAV, and Soloist. Different subfamilies exhibited distinct structural features in exons and introns. AP2 and Soloist subfamilies had more introns and were more conserved than the ERF, DREB, and RAV subfamilies. The AP2 and Soloist subfamilies in rose were mostly composed of two AP2 conserved domains, while the ERF, DREB, and RAV subfamilies are mostly composed of one AP2 conserved domain. The distribution of *RhAP2/ERF* gene family on rose chromosomes was non-random and uneven. The chromosome chr2 has the highest number of *RhAP2/ERF* genes (28), whereas the smallest number occurred on chromosome chr5 (8). Multiple *RhAP2/ERF* genes on a single chromosome are commonly found in various plant species, including *Arabidopsis* [15] and maize [25].

The main factors driving gene family amplification are segmental repeats, tandem repeats, and transposition events. Segmental duplication played an important role in the amplification of the *AP2/ERF* gene family and was the main mechanism leading to an increase in the *AP2/ERF* gene family, a finding which has also been reported in *Arabidopsis* [15] and pecan [29]. We identified 22 pairs of duplicated genes in the *RhAP2/ERF* gene family, which were all segmentation duplications. The *RhERF51* and *RhDREB22* genes have undergone multiple replicates. These findings indicate that segmental duplication contributes to the amplification of the *RhAP2/ERF* family.

The *cis*-acting elements of the promoter are important components of genes, which can reflect the potential functions and regulatory pathways of genes [22]. This study shows that the *cis*-acting elements in the *AP2/ERF* gene promoter region of rose can be mainly divided into three categories: hormone-responsive, stress-responsive, and growth- and development-related elements. Among these rose promoter sequences, light-responsive elements, abscisic acid-responsive elements, methyl jasmonate-responsive elements, and drought-induced elements are the four most common elements, a finding which is similar to the distribution pattern of *cis*-acting elements in the *AP2/ERF* family genes of the pecan, *Carya illinoinensis* [29]. Interestingly, different subfamily gene promoters may have similar response elements, such as ABA-responsive elements and methyl jasmonate-responsive elements, which are present in different subfamily gene promoters of *RhAP2/ERF*. Meanwhile, the results of protein–protein interaction analysis showed that there were extensive interactions between AP2/ERF subfamily proteins in different plants [20,22,25,30]. These indicate that AP2/ERF transcription factors from different subfamilies may be involved in the same regulatory pathway, though different subfamily genes have undergone functional differentiation.

The expression of *AP2/ERF* family genes often showed strong tissue specificity and was also affected by different durations of drought treatments. In the current study, transcriptome sequencing data and qPCR quantification of relative gene expression from rose showed that the majority of *RhAP2/ERF* members were expressed most abundantly in roots, which is consistent with the previously reported expression patterns of several *AP2/ERF* family members. In *Betula platyphylla*, *BlAP2-6* is specifically expressed in roots, [16], while, in poplar trees, *PtERF11* is highly expressed in roots [31]. In the present study, transcriptome sequencing data analysis and qRT-PCR gene expression validation in rose showed that RhDREB36 and RhERF59 may be key to the rose response to drought stress. In *Arabidopsis*, the relative expression levels of *AtDREB2A*, *AtDREB2C*, *AtERF4,* and *AtERF7* were significantly upregulated in response to drought treatment [32]; whereas, in radish, the expression levels of *RsERF003* and *RsERF039* were significantly induced by drought stress [20]. In rice, the expression levels of *OsDREB2A, OsDREB2B, OsERF48, OsERF922,* and *OsAP2-39* increased under drought stress [25]. Comparative analysis of the expression of the *AP2/ERF* gene family in rose organs and under drought stress in the current study revealed that *RhDREB44* is not only actively expressed in leaves but expression is also significantly upregulated in leaf tissue under drought stress, indicating that this gene may have dual functions; on the one hand, this gene may participate in the regulation of normal plant growth and development processes, while, on the other hand, it may be a key component of the plant drought response network. Subcellular localization studies showed that RhDREB44 is located in the nucleus, which is a prerequisite for transcription factors to exert their biological functions by changing the expression of specific genes, in response to environmental stresses, growth, and development.

## 4. Materials and Methods

### 4.1. Genome-Wide Identification and Analysis of Physicochemical Properties of the Hybrid Tea Rose AP2/ERF Gene Family

The Ensembl Plants database (http://plants.ensembl.org/Rosa_chinensis/Info/Index, accessed on 1 June 2024) contained the complete genome sequence files of the rose as well as gene annotation files. The method that was used to identify the *AP2/ERF* gene family in rose was as follows. First, the full-length amino acid sequence of Arabidopsis AtERF59 (AT1G06160.1) was used as the query sequence (https://www.arabidopsis.org/, accessed on 1 June 2024). The software TBtools (v2.085) (https://github.com/CJ-Chen/TBtools-II (accessed on 1 June 2024)) was used to preliminarily screen for protein sequences encoded by the *AP2/ERF* gene family in hybrid tea roses using protein sequences as templates, with an E value of e6 serving as the threshold [33]. After that, the AP2/ERF domain’s HMM file (PF00183) was downloaded from the Pfam protein family database (database http://pfam.sanger.ac.uk/, accessed on 1 June 2024), and the *AP2/ERF* genes were found in the rose genome database with the help of TBtools software [34,35]. Following this, candidate protein sequences lacking conserved AP2/ERF domains were deleted, and online tools Pfam (http://pfam.xfam.org/seaRhh#tabview=Table1), SMART (http://smart.embl.de/), and NCBI CDD (https://www.ncbi.nlm.nih.gov/cdd/), both of which were accessed on 1 June 2024, were utilized to the online tool WoLF PSORT (https://wolfpsort.hgc.jp/, accessed on 1 June 2024) was used to predict subcellular localization of RhAP2/ERF family members, and the TBtools software was also used to predict the physicochemical properties of RhAP2/ERF proteins, such as molecular weight and isoelectric point, with the parameters set to default values [36].

### 4.2. Phylogenetic Analysis and Chromosome Mapping of the AP2/ERF Gene Family in Hybrid Tea Rose

The entire genome sequence, protein sequences, annotation files, and protein sequences encoded by the *AP2/ERF* gene family were downloaded from the *Arabidopsis thaliana* Genome Database TAIR website (https://www.arabidopsis.org/, accessed on 5 June 2024) [37]. Multiple sequence alignments of the AtAP2/ERF family proteins in roses and Arabidopsis were performed using DNAMAN 9.0 (https://dnaman.software.informer.com (accessed on 5 June 2024)) software. A phylogenetic tree was constructed using the neighbor-joining (NJ) method, using MEGA7 (https://ngdc.cncb.ac.cn/databasecommons/database/id/572 (accessed on 5 June 2024)) software, with 1000 bootstrap replicate iterations being performed [38]. Multiple sequence alignments were performed using the ClustalW tool in MEGA7 software. Based on the gene annotation files of the rose genome, a chromosome location map of the *RhAP2/ERF* gene family was drawn up, using TBtools software.

### 4.3. Gene Structure, Promoter Sequence, and Collinearity Analysis of the AP2/ERF Gene Family in Hybrid Tea Rose

Using TBtools software, the intron and exon characteristics of *RhAP2/ERF* genes were analyzed in light of the annotation data [39]. The MEME online tool was used to identify the conserved motifs of the RhAP2/ERF proteins (https://meme-suite.org/meme/, accessed on 9 June 2024) [40]. Phytozome v13 (https://ngdc.cncb.ac.cn/databasecommons/database/id/572 (accessed on 9 June 2024)) was used to retrieve the peptide sequences of all RhAP2/ERF proteins. The NCBI CDD database (https://www.ncbi.nlm.nih.gov/cdd/, accessed on 9 June 2024) was used to identify the conserved domains in the protein structure Using TBtools software, the results of the conserved domains, conserved motifs, and gene structure were visualized, with settings like the ideal motif width of up to 10 motifs and 6 to 150 amino acid residues in a domain. The promoter sequence upstream of the coding sequence (CDS) region of each *RhAP2/ERF* gene was extracted using TBtools software, the *cis*-acting elements of *RhAP2/ERF* genes were predicted using the online tool PlantCARE (https://bioinformatics.psb.ugent.be/webtools/plantcare/html/, accessed on 9 June 2024) [41], and the light- and hormone-responsive elements were analyzed. The MCScanX tool in TBtools software (https://github.com/CJ-Chen/TBtools-II (accessed on 9 June 2024)) was used to obtain collinearity data of the *RhAP2/ERF* genes, and segmental duplication and tandem duplication genes were identified based on the rose genome data [42]. Identification of gene duplication events was based on the following criteria: if two genes were located in the same chromosomal region and were adjacent or separated by one gene, they were considered to be tandem duplicated genes; otherwise, they were considered to be segmental duplicated genes [43]. Based on the RhAP2/ERF protein and the gene CDS, the Ka, Ks, and Ka/Ks values of the *RhAP2/ERF* genes were calculated using TBtools software.

### 4.4. Protein–Protein Interaction Network Analysis of AP2/ERF Genes in Hybrid Tea Rose

A RhAP2/ERF protein–protein interaction network was constructed using the Search Tool for the Retrieval of Interacting Genes/Proteins database (https://string-db.org/, accessed on 15 June 2024), with Arabidopsis AP2/ERF proteins as templates [44]. Based on the protein interaction score (≥0.400), the number of interactions was shown to be five. The results were imported into Cytoscape software to visualize protein–protein interaction networks [45].

### 4.5. Transcriptomic Analysis of AP2/ERF Genes in Hybrid Tea Rose

RNA-sequencing (RNA-seq) data from *R. × hybrida* under drought treatment of roots, stems, leaves, flowers, or leaves for 0, 6, 9, or 24 h were downloaded from the PlantExp database (https://biotec.njau.edu.cn/plantExp/, accessed on 19 June 2024) (biological projects: SRX5973698; SRX5973700; SRX5973704; SRX6490241; SRX10603717; SRX10603718; SRX10603721; SRX10603724). The artificial drought stress experiment conducted in the current study used mature intact hybrid tea rose (*R. × hybrida*) plants as test materials, soaked in 20% PEG6000 solution for 0, 6, 9, or 24 h, and then used mature leaves taken from the middle of the plants. The expression of *AP2/ERF* genes was analyzed by transcriptome sequencing. Normalization of the reads was achieved by the FPKM (Fragments Per Kilobase of transcript per Million mapped reads) value, then |log2 FC| standardization was performed to determine significant foldchange (FC) in the expression of differentially expressed genes (DEGs), and TBtools software was used to draw an expression heat map [46].

### 4.6. Plant Material, Stress Treatments, and Tissue Collection

In this experiment, uniformly growing seedlings of the hybrid tea rose cv. Bright Smile was cultivated for 14 days under conditions of 25 °C, 16 h light/ 8 h dark photoperiod, light intensity 15 μmol·m^−1^·s^−2^, and relative humidity 40–60%. Each rose seedling was grown in a pot with a diameter of 25 cm, filled with soil composed of loam, peat, and sand (2:1:1). For tissue-specific gene expression studies, samples (0.2 g) were collected from the growing rose plants in the form of roots, stems, leaves (the first leaf under the flower), and flowers. For the drought stress study, rose cuttings (of uniform size) were soaked in 1/4 × Hoagland’s nutrient solution, pH 6.5, with (PEG6000 group) or without (control group, CK) 20% (*w*/*v*) PEG6000. Samples of leaves (the first leaf under a flower) were collected at 0, 3, 9, or 24 h after stress treatment. The samples were snap-frozen in liquid nitrogen and then stored at −80 °C until RNA was to be extracted. Three biological replicates were run for each treatment.

### 4.7. Verification by qRT-PCR of AP2/ERF Differential Gene Expression Identified by Transcriptomics of Hybrid Tea Rose Under Drought Stress

According to Yin (2008) [47] and Chi (2021) [48], the TRIzol (Thermo Fisher Scientific, Waltham, MA, USA) reagent was used to extract RNA. Using the RevertAid First Strand cDNA Synthesis Kit (Thermo Fisher Scientific, Waltham, MA, USA), reverse transcription was carried out in accordance with the directions provided by the manufacturer. Up until the time of analysis, the reverse-transcribed first-strand cDNA was packaged and stored at −80 °C. Based on the hybrid tea rose transcriptome sequencing data (Table S3), Beijing Qingke Biological Co., Ltd. (Kunming, China) synthesized primers using *LhActin* (JX826390) as the internal reference gene using Primer Premier 5.0 software (www.PremierBiosoft.com (accessed on 16 June 2024)). Using the ABI Prism 7300 Sequence Detection System (Applied Biosystems, Waltham, MA, USA), the obtained cDNA was subjected to quantitative reverse transcription PCR (qRT-PCR). The following was the composition of the reaction system (20 L): 10 mL of Hieff^®^ qPCR SYBR Green Master Mix (Yeasen Biotechnology Co., Ltd., Wuhan, China), 6.8 mL RNase-free double-distilled water, 0.6 mL each of the upstream and downstream primers, and 2 mL of cDNA were used. Following a solution curve analysis (amplification reaction conditions were forty cycles of 95 °C for two minutes, 95 °C for fifteen seconds, and 60 °C for one minute (for collection of the fluorescent signal). The amplification and dissolution curves were used to determine data reliability, and the 2^−∆∆Ct^ method was used to calculate relative gene expression levels [48].

### 4.8. Subcellular Localization

In order to determine the subcellular localization of RhDREB44 in rose, the *RhDREB* coding region was cloned, using cDNA from mature leaves of the miniature rose cv. Juice Balcony as the template. The amplified fragment was inserted between the *Sac*I and *Bam*H1 sites on the PC1300-*GFP* vector to generate the *RhDREB44-GFP* fusion construct. The plasmids PC1300-*GFP*-*RhDREB44* and the empty vector PC1300S-*GFP* were transformed into Arabidopsis protoplasts using the PEG transformation method [31]. Cells were incubated for 14–20 h in the dark at 28 °C, after which the fluorescence signal was observed using a laser confocal scanning microscope (SP8; Leica, Wetzlar, Germany).

### 4.9. Statistical Analysis

All experiments had three biological replicates and statistical analysis was performed using SPSS 18.0 (SPSS Inc., Chicago, IL, USA). Tukey’s test was performed to assess differences among treatments. Differences in means among treatments were considered significant at *p* < 0.05.

## 5. Conclusions

Using bioinformation analysis, this study identified 127 *AP2/ERF* genes from rose, belonging to five gene subfamilies. Members of each subfamily had similar gene structures, conserved motifs, and other characteristics. The gene promoter region contained elements related to response to hormones, abiotic stresses, and other functions. Gene expression exhibited strong tissue specificity, and most ERF or DREB genes responded significantly to drought stress. Among them, two *RhAP2/ERF* genes (*RhDREB36* and *RhERF59*) had high homology with Arabidopsis drought-tolerance genes (*AtDREB2A* and *AtDREB2C*), indicating that these two genes may be key to the response to drought stress. The sustained and significant expression of *RhDREB44* under drought stress, compared with the control, suggested that it may have dual functions, participating in the normal growth and development processes of plants, as well as being a key component of the plant drought response network. These results provide a theoretical basis for further exploration of the functions of *RhAP2/ERF* genes in rose growth, development, and response to abiotic stress.

## Data Availability

All available data are presented in the manuscript.

## Supplementary Materials

The following supporting information can be downloaded at: https://www.mdpi.com/article/10.3390/ijms252312849/s1.
