# Supplementary material for: Genome-Wide Identification and Expression Analysis of the AP2/ERF Transcription Factor Gene Family in Hybrid Tea Rose Under Drought Stress
Source: Int J Mol Sci. 2024 Nov 29;25(23):12849. doi: 10.3390/ijms252312849 (PMC11641635; doi:10.3390/ijms252312849)

Table S1. Characteristics of identified *RhAP2/ERF* gene gamily.

Group	Gene	ID	Length	MW	pI	Instability Index	Aliphatic Index	GRAVY	PrediRhed Subcellular Location
AP2	RhAP2-1	PRQ27885	657	72366.05	6.84	49.98	57.87	-0.676	Nucleus
	RhAP2-2	PRQ37370	553	59845.32	6.91	40.99	56.18	-0.614	Nucleus
	RhAP2-3	PRQ40849	433	48891.45	9.09	62.29	63.79	-0.789	Nucleus
	RhAP2-4	PRQ41499	832	91007.8	6.1	44.9	53.08	-0.811	Nucleus
	RhAP2-5	PRQ43438	342	38250.57	6.67	63.55	63.71	-0.674	Nucleus
	RhAP2-6	PRQ48032	535	59613.3	6.42	57.64	45.29	-0.999	Nucleus
	RhAP2-7	PRQ48269	660	73731.42	6.54	50.08	53.82	-0.803	Nucleus
	RhAP2-8	PRQ51513	577	63794.13	5.73	56.19	54.28	-0.778	Nucleus
	RhAP2-9	PRQ53670	459	52154.03	8.72	60.99	62.79	-0.792	Nucleus
	RhAP2-10	PRQ56018	401	44887.51	5.26	65.38	62	-0.805	Nucleus
	RhAP2-11	PRQ58903	457	49668.31	8.55	51.92	69.21	-0.547	Nucleus
DREB(A-1)	RhDREB1	PRQ17829	280	29944.79	5.15	57.37	71.89	-0.394	Nucleus
	RhDREB2	PRQ20759	196	21625.01	5	57.18	59.34	-0.584	Nucleus
	RhDREB3	PRQ20760	194	21504.24	5.23	71.99	64.95	-0.49	Nucleus
	RhDREB4	PRQ20769	135	15261.31	9.45	53.99	62.22	-0.6	Nucleus
	RhDREB5	PRQ20781	125	13942.8	7.99	42.55	58.72	-0.75	Nucleus
	RhDREB6	PRQ20785	194	21479.21	5.54	70.02	58.45	-0.558	Nucleus
	RhDREB7	PRQ43799	205	21867.35	5.95	52.38	68.24	-0.315	Chloroplast
	RhDREB8	PRQ43807	200	22209.44	6.19	52.8	74.2	-0.396	Nucleus
	RhDREB9	PRQ58504	211	23403.97	6.22	52.45	51.9	-0.751	Nucleus
	RhDREB10	PRQ60027	233	24448.94	5.09	49.66	63.82	-0.559	Nucleus
DREB(A-2)	RhDREB11	PRQ27125	257	28100.53	5.68	38.14	60.74	-0.498	Chloroplast
	RhDREB12	PRQ28117	149	16493.58	9.38	40.16	52.35	-0.781	Nucleus
	RhDREB13	PRQ41643	166	18569.94	9.62	44.13	48.86	-0.831	Nucleus
	RhDREB14	PRQ42810	597	68143.97	4.58	40.76	65.09	-0.842	Cytoplasm
	RhDREB15	PRQ44622	349	39434.23	6.09	50.63	58.45	-0.695	Nucleus
	RhDREB16	PRQ52905	194	21193.52	9.03	46.77	54.33	-0.832	Nucleus
	RhDREB17	PRQ59478	329	36065.44	5.79	56.37	46.6	-0.812	Nucleus
	RhDREB18	PRQ59524	384	42333.06	4.75	35.77	50.52	-0.851	Nucleus
DREB(A-3)	RhDREB19	PRQ20692	187	20815.59	9.71	59.21	73.64	-0.586	Nucleus
	RhDREB20	PRQ26750	228	25583.45	5.9	57.04	66.36	-0.857	Nucleus
	RhDREB21	PRQ26917	206	23409.48	6.24	62.82	71.07	-0.757	Chloroplast
	RhDREB22	PRQ46368	205	22908.76	9.57	66.33	69.02	-0.693	Nucleus
	RhDREB23	PRQ50461	201	22942.84	5.9	60.39	70.35	-0.629	Mitochondrial
	RhDREB24	PRQ50675	194	21750.45	7.02	68.74	71.96	-0.753	Nucleus
DREB(A-4)	RhDREB25	PRQ17831	241	27379.23	5.2	66.19	59.54	-0.872	Nucleus
	RhDREB26	PRQ17834	240	27339.17	5.29	65.19	58.17	-0.872	Nucleus
	RhDREB27	PRQ17837	241	27417.34	5.41	62.65	59.96	-0.883	Nucleus
	RhDREB28	PRQ17839	251	28672.7	5.39	57.13	60.68	-0.911	Nucleus
	RhDREB29	PRQ17841	242	27014.73	5.61	55.67	62.93	-0.834	Nucleus
	RhDREB30	PRQ18255	223	24758.23	5.13	53.5	56.55	-0.845	Nucleus
	RhDREB31	PRQ18260	179	19929.29	7.66	57.45	60.11	-0.662	Nucleus
	RhDREB32	PRQ27521	249	27028.91	5.33	63.67	63.09	-0.616	Nucleus
	RhDREB33	PRQ46665	255	27794.47	4.93	66.29	50.59	-0.755	Nucleus
	RhDREB34	PRQ51019	262	28219.2	5.2	57.34	73.4	-0.499	Nucleus

	RhDREB35	PRQ57269	277	30179.6	5.04	58.08	49.13	-0.886	Nucleus
	RhDREB36	PRQ60026	231	25247.05	5.31	55.44	56.28	-0.544	Nucleus
	RhDREB37	PRQ20745	166	17684.47	5.51	52.44	62.41	-0.576	Mitochondrial
	RhDREB38	PRQ20747	166	17829.8	5.83	50.32	68.19	-0.436	Chloroplast
DREB(A-5)	RhDREB39	PRQ21081	184	20279.95	5.48	35.13	68.97	-0.355	Chloroplast
	RhDREB40	PRQ26516	234	25620.54	5.62	61.1	63.03	-0.453	Nucleus
	RhDREB41	PRQ27727	156	17640.55	9.18	57.31	53.85	-1.053	Nucleus
	RhDREB42	PRQ47055	210	23318.64	4.88	59.44	53.52	-0.716	Cytoplasm
	RhDREB43	PRQ48034	173	18689.02	7.63	47.68	55.49	-0.646	Nucleus
	RhDREB44	PRQ21483	377	41083.68	6.19	70.02	55.46	-0.66	Nucleus
	RhDREB45	PRQ38553	402	44502.83	8.82	52.59	65.07	-0.669	Nucleus
DREB(A-6)	RhDREB46	PRQ48824	349	38754.23	5.66	62.34	63.27	-0.572	Nucleus
	RhDREB47	PRQ49830	466	52524.02	5.49	62.86	50.94	-0.898	Nucleus
	RhDREB48	PRQ59478	329	36065.44	5.79	56.37	46.6	-0.812	Nucleus
	RhERF1	PRQ16440	214	23668.42	9.34	56.34	65.7	-0.613	Nucleus
	RhERF2	PRQ16781	325	35126.79	6.06	45.45	59.72	-0.583	Mitochondrial
	RhERF3	PRQ25950	212	23205.57	10.28	63.12	52.22	-0.891	Nucleus
	RhERF4	PRQ28989	162	17542.75	9.81	46.88	75.86	-0.406	Chloroplast
	RhERF5	PRQ28991	264	28630.6	9.36	46.76	59.17	-0.695	Nucleus
	RhERF6	PRQ31188	250	26414.41	10.03	40.32	56.48	-0.682	Nucleus
	RhERF7	PRQ31970	226	24393.93	7.66	51.25	52.39	-0.754	Nucleus
ERF(B-1)	RhERF8	PRQ35742	282	31453.08	8.24	55.7	66.13	-0.658	Nucleus
	RhERF9	PRQ37369	306	33250.76	8.7	58.19	50.42	-0.665	Chloroplast
	RhERF10	PRQ39119	403	45001.16	5.11	59.02	63.2	-0.492	Nucleus
	RhERF11	PRQ39742	271	29456.84	5.17	65.22	33.17	-0.871	Nucleus
	RhERF12	PRQ40160	428	47356.66	6.56	51.35	38.76	-0.792	Nucleus
	RhERF13	PRQ41769	300	33098.41	5.9	43.65	57	-0.703	Nucleus
	RhERF14	PRQ50475	296	32219.1	9.83	36.73	69.83	-0.483	Chloroplast
	RhERF15	PRQ52662	231	25043.89	8.69	62.45	50.82	-0.688	Chloroplast
	RhERF16	PRQ53490	419	46258.85	4.62	64.49	59.98	-0.7	Nucleus
	RhERF17	PRQ23068	268	30091.47	6.91	55.85	47.8	-0.913	Nucleus
	RhERF18	PRQ26426	309	34397.68	5.41	43	53.69	-0.887	Nucleus
	RhERF19	PRQ39294	255	28391.02	6.26	55.99	68.55	-0.653	Nucleus
	RhERF20	PRQ47944	233	25420.69	8.22	46.5	57.81	-0.718	Chloroplast
	RhERF21	PRQ47962	242	26673.78	5.83	49.3	59.67	-0.643	Chloroplast
ERF(B-2)	RhERF22	PRQ47967	213	23449.16	5.61	54.51	48.54	-0.915	Nucleus
	RhERF23	PRQ47969	193	20833.27	5.22	53.01	50.57	-0.794	Chloroplast
	RhERF24	PRQ47971	181	19723.38	9.42	49.04	53.92	-0.701	Chloroplast
	RhERF25	PRQ47973	208	22422.17	8.95	38.84	60.1	-0.651	Nucleus
	RhERF26	PRQ50207	179	19580.92	8.69	51.65	56.2	-0.744	Chloroplast
	RhERF27	PRQ51725	392	43529.16	4.97	41.36	58.27	-0.74	Nucleus
	RhERF28	PRQ57559	237	25976.94	5.75	51.8	60.51	-0.73	Chloroplast
	RhERF29	PRQ16536	381	43190.39	5.32	57.44	71.34	-0.583	Nucleus
ERF(B-3)	RhERF30	PRQ37798	352	39579.08	6.12	48.6	65.14	-0.809	Nucleus
	RhERF31	PRQ40369	347	38504.66	4.75	57.34	60.95	-0.738	Nucleus
	RhERF32	PRQ58510	344	38784.55	6.07	59.82	64.62	-0.765	Nucleus
	RhERF33	PRQ16849	266	29358.38	7.15	60.54	65.71	-0.723	Nucleus
ERF(B-4)	RhERF34	PRQ16850	237	26348.86	6.65	54.55	56.92	-0.851	Nucleus
	RhERF35	PRQ24634	451	48674.27	6.84	71.31	59.31	-0.674	Nucleus

	RhERF36	PRQ25861	263	29634.18	5.56	55.56	68.59	-0.525	Nucleus
	RhERF37	PRQ25862	150	16937.64	6.76	51.23	52.13	-0.913	Nucleus
	RhERF38	PRQ25863	143	16280.95	6.75	46.69	60.21	-0.871	Nucleus
	RhERF39	PRQ25864	174	19682.93	9.84	73.29	58.39	-0.764	Nucleus
	RhERF40	PRQ25865	150	16271.89	6.75	64.75	58.67	-0.723	Nucleus
	RhERF41	PRQ25866	159	18214.64	6.85	44.99	72.96	-0.552	Nucleus
	RhERF42	PRQ29057	170	18939.32	9.6	49.46	69.53	-0.611	Nucleus
	RhERF43	PRQ29060	268	30074.52	4.86	56.45	66.68	-0.627	Nucleus
	RhERF44	PRQ36521	141	15355.82	6.14	45.06	58.23	-0.793	Nucleus
	RhERF45	PRQ36522	156	17366.07	6.63	59.91	50.19	-0.887	Nucleus
	RhERF46	PRQ36524	268	29906.85	5.04	51.61	56.87	-0.688	Nucleus
	RhERF47	PRQ39773	236	26482.7	6.16	62.22	44.28	-1.143	Nucleus
	RhERF48	PRQ41735	234	26176.9	4.52	49.35	72.09	-0.484	Nucleus
	RhERF49	PRQ49105	322	34537.07	8.6	65.38	56.43	-0.533	Nucleus
	RhERF50	PRQ49109	388	41390.46	6.47	65.29	58.17	-0.606	Nucleus
	RhERF51	PRQ17493	335	37164.01	5.05	53.46	60.87	-0.596	Nucleus
	RhERF52	PRQ17500	488	53526.62	9.71	42.02	68.16	-0.731	Chloroplast
ERF(B-5)	RhERF53	PRQ24820	323	36175.81	5.02	55.62	82.04	-0.456	Nucleus
	RhERF54	PRQ34841	266	30143.48	5.43	61.94	67.44	-0.66	Nucleus
	RhERF55	PRQ53467	357	40131.26	5.69	54.52	80.78	-0.42	Chloroplast
	RhERF56	PRQ58378	301	34129.09	5.05	59.45	75.42	-0.668	Nucleus
	RhERF57	PRQ60332	364	40765.55	5.1	60.57	62.72	-0.632	Nucleus
	RhERF58	PRQ18313	341	38196.04	7.77	56.69	67.48	-0.575	Nucleus
	RhERF59	PRQ18316	292	29878.94	4.96	46.67	68.9	-0.261	Nucleus
	RhERF60	PRQ57372	273	29767.01	4.78	58.06	67.51	-0.475	Nucleus
	RhERF61	PRQ57373	213	23776.62	6.54	66.15	69.67	-0.694	Nucleus
ERF(B-6)	RhERF62	PRQ57374	239	26514.36	5.56	58.59	60.96	-0.644	Nucleus
	RhERF63	PRQ57376	217	23598.04	6.39	65.42	57.14	-0.687	Nucleus
	RhERF64	PRQ57377	204	22842.3	6.99	63.93	59.85	-0.843	Nucleus
	RhERF65	PRQ59748	286	31974.5	5.64	49.76	67.87	-0.735	Nucleus
	RhERF66	PRQ59749	293	32092.88	7.02	65.61	71.02	-0.52	Nucleus
	RhERF67	PRQ59750	214	24136.27	4.75	81.83	52.52	-1.014	Nucleus
RAV	RhRAV	PRQ40417	366	40648.7	9.23	47.45	68.47	-0.702	Nucleus
Soloist	RhSoloist	PRQ44589	404	44804.07	5.4	56.27	76.98	-0.52	Nucleus

Table S2. Intra segmental replication of AP2/ERF homologous pairs in roses.

Paralogous AP2/ERF Pairs	chr.Location	Duplication Type	Ka	Ks	Ka/Ks
RhAP2-11<br>RhAP2-5	Chr3<br>Chr1	Segmental	0.4172	1.3811	0.3021
RhERF60<br>RhERF59	Chr1<br>Chr7	Segmental	0.4830	4.2057	0.1148
RhERF56<br>RhERF51	Chr1<br>Chr7	Segmental	0.5025	2.3756	0.2115
RhDREB10<br>RhDREB29	Chr1<br>Chr7	Segmental	0.5768	2.4445	0.2360
RhERF57<br>RhERF51	Chr1<br>Chr7	Segmental	0.4099	NaN	NaN
RhDREB22<br>RhDREB24	Chr2<br>Chr2	Segmental	0.2455	1.9345	0.1269

RhAP2-9<br>RhAP2-3	Chr2<br>Chr4	Segmental	0.3192	1.9730	0.1618
RhERF16<br>RhRAV	Chr2<br>Chr4	Segmental	0.2629	NaN	NaN
RhERF15<br>RhERF7	Chr2<br>Chr5	Segmental	0.2971	2.1093	0.1408
RhAP2-7<br>RhAP2-1	Chr2<br>Chr6	Segmental	0.2551	1.2679	0.2012
RhDREB43<br>RhDREB41	Chr2<br>Chr6	Segmental	0.2704	1.4919	0.1812
RhDREB33<br>RhDREB32	Chr2<br>Chr6	Segmental	0.3515	1.3864	0.2535
RhDREB22<br>RhDREB20	Chr2<br>Chr6	Segmental	0.3276	1.8845	0.1738
RhDREB23<br>RhDREB21	Chr2<br>Chr6	Segmental	0.2883	2.8357	0.1017
RhERF21<br>RhERF17	Chr2<br>Chr6	Segmental	0.5208	2.0939	0.2487
RhDREB46<br>RhDREB44	Chr2<br>Chr7	Segmental	0.2996	1.1785	0.2542
RhERF19<br>RhERF18	Chr4<br>Chr6	Segmental	0.6303	3.1163	0.2023
RhERF44<br>RhERF39	Chr4<br>Chr6	Segmental	0.3282	NaN	NaN
RhERF45<br>RhERF37	Chr4<br>Chr6	Segmental	0.3742	1.7401	0.2151
RhERF46<br>RhERF36	Chr4<br>Chr6	Segmental	0.3186	NaN	NaN
RhERF47<br>RhERF33	Chr4<br>Chr7	Segmental	0.4411	1.7849	0.2472
RhERF31<br>RhERF29	Chr4<br>Chr7	Segmental	0.7010	NaN	NaN

Table S3. Primers of RhAP2/ERF gene needed for fluorescence quantification

Primer name	Sequence(5′-3′)	Annealing Temperature（℃）
Rhactin-qF	5' CCCCARhCAATCCCAAGGCAA 3'	55.5
Rhactin-qR	5' CGGAAGTCCAGCACAATACCA 3'	
RhERF5-qF	5' CCGAGAACCGAGAAAACCGCCA 3'	52.1
RhERF5-qR	5' GCCTCTTCCTCACTCCCCTAAA 3'	
RhERF6-qF	5' CCCCTCGGGCGACCCATCTTTT 3'	53.3
RhERF6-qR	5' GGCGGCTGCCTTGACGCTTTTG 3'	
RhERF7-qF	5' CGGACCCAAAGCCAAGACCAAT 3'	51.2
RhERF7-qR	5' CCCGTAACCCGACCCGAACAGC 3'	
RhERF15-qF	5' GGCAGCCGCAGCCCCGAAAACG 3'	56.3
RhERF15-qR	5' GAAGGTCCCGAGCCAGACACGC 3'	

RhERF66-qF	5'	GGGTGTGGAGAGTAATAAAGCG	3'	52.3
RhERF66-qR	5'	AGATAATGGTGACAAAGGAGGC	3'	
RhERF67-qF	5'	CCTCAACCCGAAAACCGAACCC	3'	58.6
RhERF67-qR	5'	TCTCCGCCGCAAACTTGCCCCA	3'	
RhDREB44-qF	5'	CCGCCTCAATTTCCCTCATTTG	3'	57
RhDREB44-qR	5'	GCTTCCCTGCCTTTCCCTGTTT	3'	
RhRAV-qF	5'	TGGCGGTTCCGGTACTCCTACT	3'	56.3
RhRAV-qR	5'	TGCTCCGTGCCTTCCAATCTAT	3'	
RhDREB36-qF	5'	GTACCCATTTGGTTCGGATTTG	3'	55.1
RhDREB36-qR	5'	CTTCTTCCTTCCCGCCCTCTTC	3'	
RhERF59-qF	5'	GAGCTCCAGCTTCAGCAGCTTG	3'	56
RhERF59-qR	5'	ACGGCGTCTCTTAGAACACCGT	3'	
RhDREB44-qF	5'	CCGCCTCAATTTCCCTCATTTG	3'	57
RhDREB44-qR	5'	GCTTCCCTGCCTTTCCCTGTTT	3'	
RhERF17-qF	5'	AACTCTCTCTTCCCTTCCTACC	3'	53.8
RhERF17-qR	5'	ATTCTTCTTCAGCCTCTTCTTG	3'	
RhERF2-qF	5'	TCCAACACACTTCCTATGCCTA	3'	53.4
RhERF2-qR	5'	CACCACCTCCCATTGCTCCTTC	3'	
RhERF35-qF	5'	CAACAACAACCCCAGCCGACGA	3'	55.2
RhERF35-qR	5'	AGGATGGCAATGCGGAAGACAC	3'	
RhERF50-qF	5'	AGGAAATGAGACCGACCAAACT	3'	52.5
RhERF50-qR	5'	ACCACCACTACTACCACCAGCA	3'	
RhDREB37-qF	5'	GAAGGGAGTGCGTCAAAGAAAA	3'	56.5
RhDREB37-qR	5'	ACACGGCAAGACTGTAGGAAAG	3'	

Figure S1. Types of *cis-elements* in the *RhAP2/ERF* gene promoter of *Rosa hybrida*

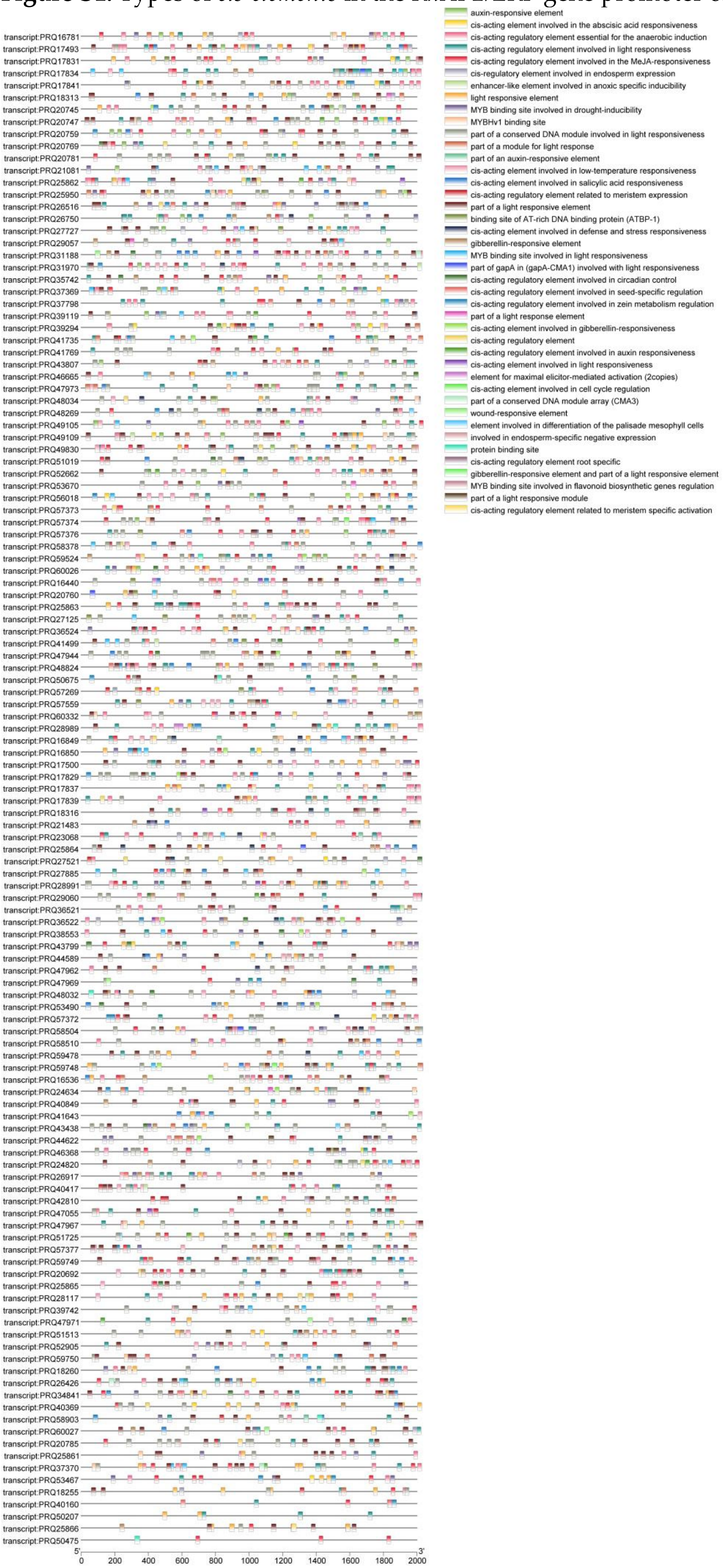

Supplement: Supplementary file 1 [file ijms-25-12849-s001.zip › ijms-3268224-supplementary.pdf]
